# Do people with intellectual disabilities understand their prescription medication? A scoping review

**DOI:** 10.1111/jar.12643

**Published:** 2019-07-24

**Authors:** Megan V. A. Smith, Danielle Adams, Claudia Carr, Silvana E. Mengoni

**Affiliations:** ^1^ Centre for Health Services and Clinical Research University of Hertfordshire Hatfield UK; ^2^ Independent Specialist Mental Health Pharmacist UK; ^3^ Hertfordshire Law School University of Hertfordshire Hatfield UK

**Keywords:** decision making, intellectual disability, medicine, prescriptions

## Abstract

**Background:**

People with intellectual disabilities are more likely to experience poor health than the general population and are frequently prescribed multiple medications. Therefore, it is important that people with intellectual disabilities understand their medication and potential adverse effects.

**Method:**

A scoping review explored people with intellectual disabilities' knowledge of prescription medications, their risks and how medication understanding can be improved.

**Results:**

Ten journal articles were included. People with intellectual disabilities often lacked understanding of their medication, including its name, purpose and when and how to take it. Participants were often confused or unaware of adverse effects associated with their medication. Information was sometimes explained to carers rather than people with intellectual disabilities. Some interventions and accessible information helped to improve knowledge in people with intellectual disabilities.

**Conclusion:**

There is a need for accessible and tailored information about medication to be discussed with people with intellectual disabilities in order to meet legal and best practice standards.

## 1 INTRODUCTION

People with intellectual disabilities are more likely to experience poor health than the general population (Heslop et al., 2014; Krahn, Hammond, & Turner, 2006; van Schrojenstein Lantman‐de Valk & Walsh, 2008). Healthcare needs for people with intellectual disabilities can be complex, with a higher burden of multimorbidity and long‐term conditions such as epilepsy, psychosis, dementia and heart failure (Carey et al., 2016; Cooper et al., 2018; NHS Digital, 2016) and correspondingly greater medication use than in the general population (Doan, Lennox, Taylor‐Gomez, & Ware, 2013). However, there is limited research evidence about how people with intellectual disabilities understand their medication, and what support can be provided to maximize this.

Health service use is greater for people with intellectual disabilities compared to the general population, including a higher number of primary care consultations per year (Carey et al., 2016) and a higher rate of hospital admissions (Dunn, Hughes‐McCormack, & Cooper, 2017; Glover & Evison, 2013), yet there is some evidence that healthcare providers may be less likely to follow best practice when treating people with intellectual disabilities (Dunn et al., 2017). Quality and Outcome Framework (QOF) indicators are used in the UK National Health Service (NHS), and they aim to measure outcomes that reflect the quality of healthcare (NHS Confederation, 2018; NICE, 2019). Cooper et al. (2018) found that many QOF indicators were significantly less likely to be met in primary care for people with intellectual disabilities and long‐term conditions such as epilepsy, diabetes and hypertension, compared to the general population. Compared to the general population with the relevant long‐term condition, this included fewer people with intellectual disabilities and diabetes having a HbA1C record in the previous 15 months, and fewer people with asthma and intellectual disabilities having an asthma review in the previous 15 months.

An effective and optimized medication regime is an integral part of managing health conditions. It is important for patients to understand the information about their medication to facilitate the management of health conditions. As part of this, effectively informing patients about the risks of adverse effects is essential for them to make informed decisions about their medicine‐taking and adhere to their treatment (Knapp, Raynor, & Berry, 2004). In the general population, prescription medication instructions and warnings are often complex and, as a consequence, may not be fully understood resulting in misuse and adverse effects (Davis, Wolf, Bass, Middlebrooks, et al., 2006; Davis, Wolf, Bass, Thompson, et al., 2006; Wolf, Davis, Shrank, Neuberger, & Parker, 2006; Wolf, Davis, Tilson, Bass, & Parker, 2006). In particular, studies have shown that people with low literacy levels often have difficulty both understanding and reading labels on medication (Davis, Wolf, Bass, Middlebrooks, et al., 2006; Wolf, Davis, Tilson, et al., 2006), which may predispose them to increased risk of experiencing adverse effects. The terms “adverse effects” and “side effects” of medicines are frequently used interchangeably. However, an “adverse effect” is an undesirable harmful effect resulting from a medicine, for example, ibuprofen increasing risk of peptic ulceration and olanzapine increasing the risk of constipation. Whereas a "side effect" is an effect secondary to the main or therapeutic effect of a medicine which may not necessarily be harmful and can be desirable in some circumstances. For the purposes of this scoping review, the present authors will refer to “adverse effects” with the exception of when reporting studies which have used different terminology.

There is often a high usage of prescription medications in people with intellectual disabilities. In a review of a general practice database, Straetmans, Schrojenstein Lantman‐de Valk, Schellevis, and Dinant (2007) found that prescription medications were received by 75% of people with intellectual disabilities compared to 59% of people without intellectual disabilities, and people with intellectual disabilities received 5.4 repeat prescriptions per year compared to 1.6 by those without intellectual disabilities. More recently, research has focused on the high levels of prescriptions of psychotropic medication for people with intellectual disabilities. Doan et al. (2013) found 35% of adults living with intellectual disabilities were prescribed psychotropic medications, mainly antipsychotics. In particular, studies suggest people with intellectual disabilities may be inappropriately prescribed psychotropic medication for the management of behaviours that challenge (Deb & Unwin, 2007; Holden & Gitlesen, 2004; Matson & Neal, 2009; Sheehan et al., 2015; Singh & Matson, 2009). A UK population‐based cohort study reported that 49% of 33,016 people with intellectual disabilities were prescribed psychotropic medication yet only 21% had a record of mental illness and 25% had a record of challenging behaviour (Sheehan et al., 2015). It has been estimated that, in England, up to 35,000 adults with intellectual disabilities are being prescribed a psychotropic medicine without appropriate clinical justification (NHS England, 2018).

People with intellectual disabilities may be at increased risk of poor medication knowledge as they often experience additional challenges in health‐related communication due to factors including lack of accessible information, poor communication skills of healthcare professionals and communication, and adaptive and cognitive difficulties associated with intellectual disabilities (Marks, Sisirak, & Hsieh, 2008; Mastebroek, Naaldenberg, Lagro‐Janssen, & van Schrojenstein Lantman de Valk, 2014). Indeed, Strydom, Forster, Wilkie, Edwards, and Hall (2001) found that over half of people with intellectual disabilities interviewed were unable to read the label on their antipsychotic medicine and 86% did not know, or only knew one of, the adverse effects of their medication.

Patient‐centred practice is incorporated into guidelines for healthcare provision, including for people with intellectual disabilities. Patient‐centred practice aims to focus care on the needs of the person receiving the care rather than the needs of the service (Royal College of Nursing, 2016). The General Medical Council (GMC) is a body that sets standards for doctors in the UK and GMC guidance (c) emphasizes the importance of the doctor–patient relationship where a dialogue is created between the parties together with an exchange of views to enhance the patient's decision‐making process. All organizations providing NHS care and/or publicly funded adult social care are legally required to follow the Accessible Information Standard (AIS;NHS England, 2016). This involves taking steps to ensure that the individual, including those with intellectual disabilities, receives information in an accessible format and receives any communication support they may need, and applies to discussions around medication (NHS England, 2017).

According to the Supreme Court case of Montgomery v Lanarkshire Health Board (Scotland) (2015), where a person has capacity, healthcare professionals are under a duty “to take reasonable care to ensure that the patient is aware of any material risks involved in any recommended treatment, and of any reasonable alternative or variant” (para. 87). The judgement explains that a material risk is one that a “reasonable person in the patient's position” (para. 87) would consider significant or that the doctor is or should be aware that the patient would deem is significant (Barnett & Carr, 2018; Sokol, 2015). Where people with intellectual disabilities are concerned*,* the Montgomery case applies to those with mental capacity only, that is, able to make decisions for themselves about treatment. Therefore, the need to ensure that people with intellectual disabilities understand their medication, and its potential risks, is crucial in order to promote good health outcomes and to ensure that healthcare providers are complying with legal requirements.

### 1.1 Aims

Prescribed medications to manage health problems have the potential to cause short‐term and long‐term adverse effects. An individual with intellectual disabilities, just as those without intellectual disabilities, should be enabled to take an active part in the decision‐making process. Empowering people with intellectual disabilities to be equal stakeholders in relation to their medicines and encouraging dialogue about medicines could lead to an improvement in therapeutic relationships, better access to healthcare, improved adherence to pharmacological interventions and consequently a reduction in morbidity and mortality (Adams & Carr, 2017). There has been limited research exploring the understanding of medication within the intellectual disability population; therefore, this scoping review aimed to identify and analyse studies, which explored people with intellectual disabilities' understanding of prescription medications and the risks associated with these, along with how to improve understanding.

## 2 METHODS

### 2.1 Scoping review

Scoping reviews aim to map all of the relevant literature in a specific area of interest and consequently help to identify any gaps in existing research (Arksey & O'Malley, 2005). This approach was adopted as existing knowledge of the literature suggested a lack of previous research exploring people with intellectual disabilities' understanding of risks associated with their prescription medication. This review utilized Arksey and O'Malley's (2005) framework for scoping reviews which involves the following five stages: (1) identifying the research question; (2) identifying the relevant studies; (3) study selection; (4) charting the data; (5) collating, summarizing and reporting the results.

### 2.2 Stage 1: Identifying the research question

Three research questions directed this scoping review to address current gaps in the literature:

1. What understanding do people with intellectual disabilities have of their prescription medications?
2. What understanding do people with intellectual disabilities have of the risks (i.e., risk of an adverse effect) associated with their prescription medications?
3. How can people with intellectual disabilities' understanding of prescription medication be improved?

### 2.3 Stage 2: Identifying relevant studies

Between the 18 and 20 January 2017, searches of the following electronic databases were carried out; PubMed, Scopus, CINAHL, OpenGrey, the Cochrane database of systematic reviews and Web of Science. The search was repeated on 9 May 2018 to search the databases for papers published between 1 January 2017 and 31 December 2017. Additionally, references from the identified articles were screened to identify any further relevant material. As a result of the June 2011 Panorama exposure of the ongoing abuse at Winterbourne View, practice and policy regarding intellectual disabilities are changing. At Winterbourne View, a private hospital in England, it was found that staff routinely mistreated and abused people with intellectual disabilities in their care, leading to criminal convictions. In December 2012, the Department of Health (2012) Review “Transforming Care: A National Response to Winterbourne View Hospital” recommended that people with intellectual disabilities should be supported in community settings and not in long‐stay hospitals. Following concerns regarding the overuse of psychotropic medicines, it is recommended that medication is frequently reviewed by the healthcare team. These changes together with the strong focus on person‐centred care, planning and advocacy have led to a shift in policy regarding supporting people with intellectual disabilities (Department of Health, 2012). Literature published prior to 2011, therefore, may not be relevant to current practice and so this scoping review only included literature published from 2011 onwards. Further details regarding the search terms used are provided in Table 1.

**Table 1:** Search terms

Search term 1	Search term 2	Search term 3
*Search operator*	*AND*	*AND*
Intellectual disability	Medications	Side effects
Learning disability	Prescriptions	Risks
Learning disorders	Drugs	Knowledge
Learning difficulties	Pharmaceutical preparations	Understanding
	Prescription	Decision making
	Medicine	Consent
	Medicines	Information
	Pharmacological interventions	Informed consent
		Health education
		Health literacy
		Adverse effects

### 2.4 Stage 3: Study selection

Studies were included if they (a) reported on people with intellectual disabilities' understanding of prescription medication from the perspective of people with intellectual disabilities or others (e.g., family members, paid caregivers) or (b) aimed to improve people with intellectual disabilities' understanding of prescription medication. The search was restricted to published and peer‐reviewed journals and studies were included if published between 2011 and 2017.

Studies were excluded from this scoping review if they met any of the following criteria:

1. only reported understanding of non‐medication treatments such as surgery or psychological therapy
2. were not written in the English language (due to the time and cost involved in translating them to English)
3. only reported the medication knowledge of family or paid carers
4. were an animal study
5. were a review article.

Retrieved records from the database searches were extracted and imported in to EndNote, where duplicates were removed. Two researchers independently screened the titles and abstracts based on the inclusion/exclusion criteria described earlier. Following the initial screening, full‐text articles were read by both reviewers to make a final decision of inclusion. Any disagreements about the inclusion of the articles were resolved by discussion with a third member of the research team. The reference lists of all included articles and all excluded relevant review articles were examined to ensure that all relevant and eligible studies had been identified.

### 2.5 Stage 4: Charting the data

A data extraction sheet was developed in Excel, and relevant articles were charted using the following column headings:

1. Author(s), year of publication, study location
2. Type of publication and study
3. Main purpose of study
4. Methodological approach
5. Participant characteristics and sample size
6. Methodology
7. Setting
8. Intervention (if applicable)
9. Data collection and data analysis
10. Main findings.

Two researchers piloted the data extraction chart on one study and one of the researchers independently extracted data from each of the remaining studies.

### 2.6 Stage 5: Collating, summarizing and reporting the results

All extracted data from the included articles were summarized and tabulated by a member of the research team. In line with Arksey and O'Malley (2005), we presented our narrative account in two ways. Firstly, we reported the nature and distribution of the studies included, for example study design, country, settings and participant group. Secondly, we organized the literature according to the following themes which were drawn from the research questions: understanding of prescription medications, understanding of prescription medication risks and how to improve understanding of prescription medication in people with intellectual disabilities.

## 3 RESULTS

### 3.1 Included studies

**Figure 1:**
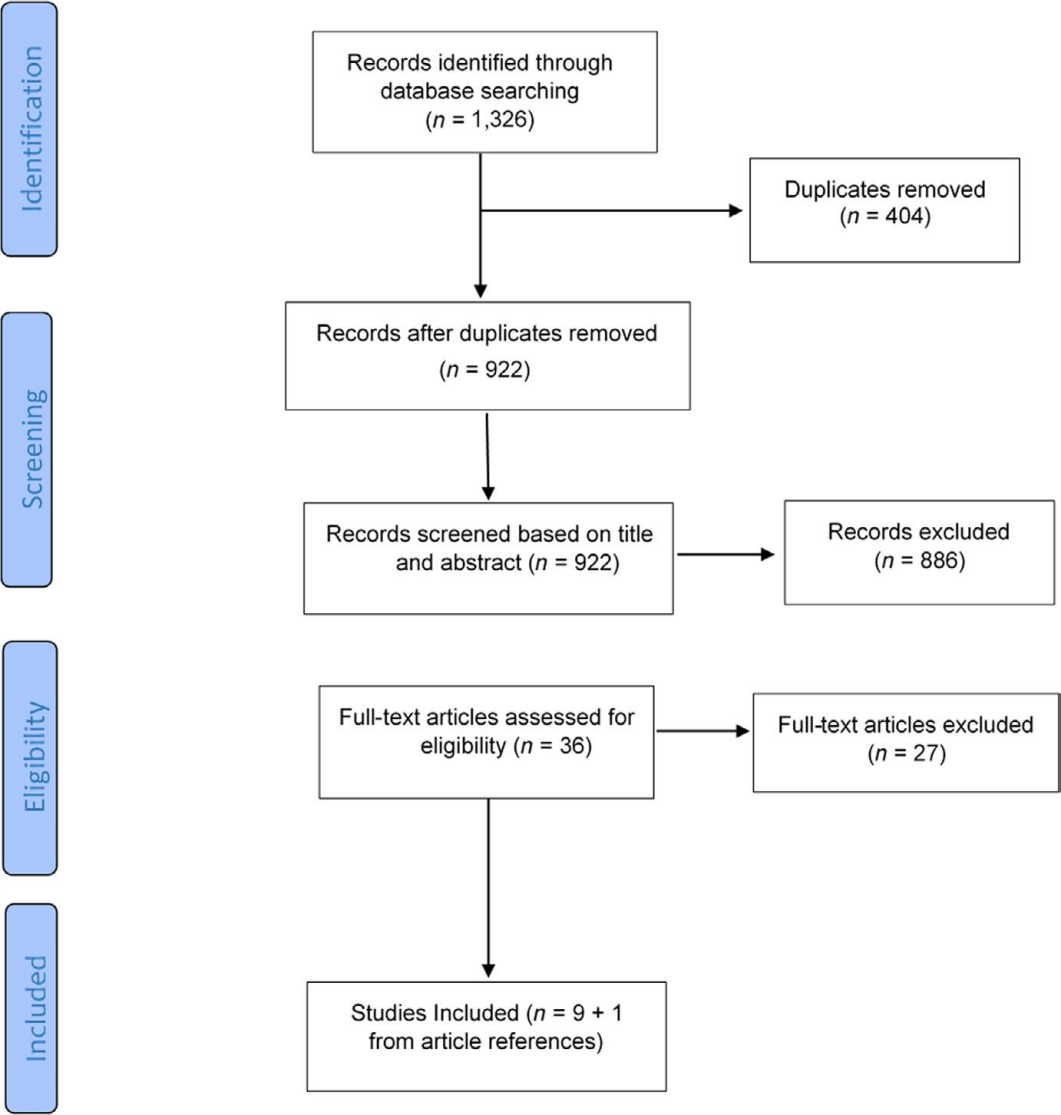
Flow chart of studies from identification to inclusion

The searches in 2017 and 2018 are presented together as the search methodology was the same. Initial searching of the aforementioned electronic databases provided 1,326 records. Four hundred and four duplicates were removed leaving 922 records to screen for relevance based on title and abstract. Based on title and abstract screening, 886 records were excluded. Thirty‐six full‐text articles were retrieved and assessed by the researchers for inclusion. Twenty‐seven studies were excluded. A total of nine studies met all inclusion criteria and were subsequently included in the review. When screening the references of the nine included studies, one article (Dysch, Chung, & Fox, 2012) was found to fit the inclusion criteria and was therefore also included within the review. An overview of the study selection process is provided in Figure 1.

### 3.2 Study characteristics

All identified articles reported completed studies. Characteristics of the included articles are summarized in Table 2. Study designs included a medical records search (Davis et al., 2014), quantitative studies (Beacroft & Dodd, 2010; Huneke, Gupta, Halder, & Chaudhry, 2012), qualitative studies (Davis et al., 2016; Dysch et al., 2012; Fish, Hatton, & Chauhan, 2017; Walmsley et al., 2016), a case study (Flood & Henman, 2015) and two pre‐/post‐test interventions (Ferguson & Murphy, 2014; Sheehan, Rochester, Hafesji, Kyambadde, & Gravestock, 2017). The intervention in Ferguson and Murphy's study consisted of three training sessions including information on the function of medication, possible side effects, risks, benefits and alternatives to medication. Sheehan et al.'s intervention was a psychotropic medication education group that took place once a week for eight weeks discussing indications, how medication could help, possible side effects and how to manage them, and where to seek further information.

**Table 2:** Overview of medication knowledge amongst people with intellectual disabilities

Author, year	Study design	Study aim(s)	Participants	Methodology	Findings
Beacroft & Dodd, 2010	Audit using interviews	To investigate pain recognition and management in people with intellectual disabilities	*N* = 40; people aged 18 and over with mild–moderate intellectual disabilities with good verbal abilities (as rated by service manager)	Interviews structured by a 40‐item questionnaire containing both open and closed questions with sections on views about pain medication	30% would not take medicine if in pain, and when asked why, there was confusion and concern around whether the pain medication would react badly with their regular medication 18 participants (45%) did not know what medication they took
Davis et al., 2014	Retrospective medical record audit	To describe respiratory medication use, prevalence of asthma and asthma management practices	*N* = 125; accessible records for patients (aged 18+) with intellectual disabilities and asthma whom attended a specialized Australian clinic. 107 of which were prescribed asthma medications	Electronic and hard copy health records were analysed quantitatively and qualitatively including content analysis of comments regarding use of respiratory medications from the hard copy records to identify any issues relating to inhalers	Inhaler use was identified as an issue, particularly inhaler technique Respiratory physicians, asthma educators, GPs and pharmacists were identified as important in training people with intellectual disabilities in inhaler use Ability to use inhalers was not reported for all participants. Where this was commented on, records showed one patient was unsure how to use their inhaler, and other patients were noted to want advice on inhaler use or need constant reminding to use their inhaler
Davis et al., 2016	Qualitative study	To explore the level of understanding of inhaled asthma medication use of people with intellectual disabilities, in the context of asthma self‐management	*N* = 17; adults with intellectual disabilities, diagnosed with asthma and who self‐administered asthma medication	Semi‐structured face‐to‐face interviews based on thematic framework approach including questions about understanding asthma medications.	Three themes emerged: o understanding of their illness and need for medication o self‐management o autonomy versus dependence Participants showed an awareness of their diagnosis and what their different medications did Most participants related not using medication to negative health consequences such as hospitalization and difficulty breathing A majority of participants did not know what the potential risks or side effects of inhalers were
Dysch et al., 2012	Qualitative study	To explore the subjective experiences and perceptions of people with intellectual disabilities and diabetes	*N* = 4; Adults (18+) with mild intellectual disabilities, capacity to consent and with a diagnosis of type 1 or 2 diabetes	Semi‐structured interviews including questions about perceptions of health and being healthy, what diabetes meant to the person, how it affected their life, received support and what they thought might happen in the future.	Participants could use language associated with their illness, which was interpreted as showing understanding of diabetes One participant stated knowing the consequences of not taking their medication properly When discussing administering medication by injection, one participant stated “I know I've got to do it so save my life”
Ferguson & Murphy, 2014	Pre‐/post‐test intervention	To investigate the capacity of people with intellectual disabilities to make decisions about their medication and to evaluate whether the provision of medication training sessions would increase this capacity	*N* = 28; adults (18+) with mild to moderate intellectual disabilities currently taking metformin, haloperidol or sodium valproate medications or on multiple medications	Participants were split into groups depending on medication and all received three training sessions tailored to their medications. The Adapted Assessment of Capacity Questionnaire (A‐ACQ) was adapted to focus on medication taking. The adapted A‐ACQ and British Picture Vocabulary Scale‐II (BVPS‐II; assesses language comprehension) measures were taken at baseline, pre‐ and post‐intervention	Highly significant positive correlations between the BPVS‐II scores and A‐ACQ scores at baseline assessment, suggesting a relationship between verbal ability and capacity The A‐ACQ scores increased significantly following the intervention
Fish et al., 2017	Mixed‐methods study using questionnaires	To gauge the level of information currently being provided to people with intellectual disabilities from their GP and pharmacist about medications, and the requirements of people with intellectual disabilities for information related to their medication	*N* = 58; intellectual disabilities self‐advocates at the North West Self‐Advocacy conference	Easy‐read questionnaire (accompanied by pictures) completed by people with intellectual disabilities to explore their views on information given by health care professionals regarding medication	The most frequent themes were that the information was not always accessible to participants. 24% reported receiving no information regarding medication at all When information was provided, it was instructional, for example, dosage and when to take the medicine 66% of people wanted easy‐read leaflets and 17% wanted pictures or diagrams to present information
Flood & Henman, 2015	Case study using an interview and medical record review	To determine the views and knowledge of a person with intellectual disabilities about medication use	*N* = 1; a man with mild intellectual disabilities and diabetes aged between 30 and 40 years	Interview analysed with a Grounded Theory approach. The participant's medication items were also reviewed	The participant appeared to be responsible for his own diabetes self‐care. He described how he administered insulin and took his tablets only because he does not “want to die”
Huneke et al., 2012	Quantitative audit using questionnaires	To evaluate current practice concerning consent to treatment in patients with intellectual disabilities against best practice guidelines and to see whether these patients are given enough understandable information, to make informed decisions about their medication	*N* = 45; adult patients with intellectual disabilities living in supported accommodation. Eight had capacity to consent. medical treatment according to staff	Participants received easy‐read format questionnaires via post and, with the help of their support worker were asked to complete and return the survey The questionnaires included Picture Communication Symbols (PCS), assessing patients' knowledge regarding their medication including possible adverse effects and consequences of not taking the medication. Patients' medication knowledge was calculated	Those with capacity showed good knowledge of the purpose of their medication, dose schedule, advantages of the treatment, and consequences of not taking the medication and contraindicated foods and drinks Participants with capacity had poor knowledge of the proposed duration of their treatment and the possible disadvantages and names of their medications Those without capacity had less knowledge of medications than those identified as having capacity. Knowledge of medication names, duration of treatment and possible disadvantages were particularly poor
Sheehan et al., 2017	Pre‐/post‐test intervention	To establish and evaluate a psychotropic medication education group for men with intellectual disability on a secure psychiatric ward	*N* = 6; men with intellectual disabilities on a secure psychiatric ward with a range of psychiatric diagnoses at various stages of recovery	People with intellectual disabilities participated in a psychotropic medication education group, once weekly over 8 weeks. Knowledge and confidence with prescribed medication were assessed by self‐report and by a test of medication knowledge (20 true or false statements) pre‐ and post‐ intervention	There was no statistically significant difference between pre‐ and post‐group scores in the psychotropic medication knowledge test (*t* = 0.250; *p* = 0.813) At baseline, all participants strongly agreed with the statements “I know enough about my medication” and “Medication is an important part of my treatment.” There were few differences in the self‐report measures post‐intervention 5 out of 6 participants “strongly agreed” they had met their personal objectives by attending the group, the remaining participant was “not sure”
Walmsley et al., 2016	Qualitative study	To explore how the reproductive capacity of women with intellectual disabilities is managed and the associated processes of contraceptive decision making	*N* = 19; women with intellectual disabilities who were users of specialist intellectual disability services and using, intending to use or had used contraception	Face‐to‐face interviews were conducted using a questionnaire in easy‐read format with illustrations	Several participants had no understanding of contraception as a means to prevent pregnancy ‐ only 2 were well informed Few women had made an informed choice about the type of contraceptive to use Most reported the decision being made for them Several women reported that the contraception prescribed to manage periods was ineffective

Of the included articles, eight were conducted in the United Kingdom (Beacroft & Dodd, 2010; Dysch et al., 2012; Ferguson & Murphy, 2014; Fish et al., 2017; Flood & Henman, 2015; Huneke et al., 2012; Sheehan et al., 2017; Walmsley et al., 2016) and two in Australia (Davis et al., 2016, 2014). All ten articles had been completed and published.

#### 3.2.1 Participants

All articles discussed adults (18+) with intellectual disabilities. Data were collected from participants with intellectual disabilities and asthma in two of the included studies (Davis et al., 2016, 2014). Two studies involved participants with intellectual disabilities and diabetes (Dysch et al.,  2012; Flood & Henman, 2015). One study interviewed females with intellectual disabilities who were using, intended to or had used contraception (Walmsley et al., 2016). The other five studies included in this scoping review were not constrained to a particular condition (Beacroft & Dodd, 2010; Ferguson & Murphy, 2014; Fish et al., 2017; Huneke et al., 2012; Sheehan et al., 2017). The studies were conducted in a range of settings including day services and supported living (Beacroft & Dodd, 2010; Huneke et al., 2012), secure psychiatry wards (Sheehan et al., 2017) and a self‐advocacy conference (Fish et al., 2017).

### 3.3 Knowledge of prescription medications

There was a range of findings about the medication knowledge of people with intellectual disabilities, but the majority of articles found evidence that many people with intellectual disabilities did not fully understand their prescription medication, including medication names, its purpose, when and how to take it and how to store it.

#### 3.3.1 Purpose of medication

Two of the included articles presented findings showing good levels of knowledge amongst people with intellectual disabilities regarding prescription medication, including what medication they were taking and why. Participants in Sheehan et al.'s (2017) education study rated their knowledge of and comfort with medication before and after participating in a psychotropic medication education group. At baseline and post‐intervention, all participants reported strongly agreeing with knowing enough about their medication and that medication is an important part of their treatment.

Davis et al. (2016) carried out 17 semi‐structured interviews to explore the level of understanding of prescription inhalers in people with intellectual disabilities and asthma. They found that most participants were aware of their diagnosis and what their medication did for them, for example, “relax the airway when it's wheezy.” Most participants were also able to link not taking medication to negative health consequences, for example, “if I don't take that, I feel a bit funny when I start walking. I get choked up a bit,” “if I didn't use it I'd probably be in hospital” and “if I don't take this one, I get sick.”

Two studies found more variable medication knowledge, but with some notable cases of good understanding. In Dysch et al.'s (2012) study, one participant expressed an understanding of diabetes medication “so they have insulin injections to actually um, grasp more insulin inside themselves” and one participant was able to identify early warning signs that she had not taken her medication “I can tell when I've not had the tablets and stuff. Sometimes I feel hungry or empty. And I've got to have something to make myself well.” Some participants expressed understanding the consequences of not taking their medication “I know the consequences…where you might lose a limb or something like that, which people could do if they don't take their medication properly” and “I know I've got to do it to save my life.” Walmsley et al. (2016) found that of the 19 women with intellectual disabilities they interviewed about contraception use, two women were well informed, with one expressing knowledge of when you should be on contraception and how to get it “if you are in a relationship, you see a specialist, get sensible contraception advice and also talk to a specialist about the right contraception with your doctor….”

The 19 participants in Walmsley et al. (2016) were all using, intending to or had used contraception in the past, yet in some cases there was little knowledge and understanding about what contraception was and why it would be used, for example, when asked “contraception—do you know what it means?” one participant responded “your health? No, not much.” The authors report that several participants also appeared to have no understanding of contraception as a pregnancy preventative.

Beacroft and Dodd's study (2010) found poor knowledge of the purpose of medication. They found eighteen of 40 participants (45%) did not know what medication they took and although 53% reported that they knew why they were taking prescription medications, they did not know the name of their medication(s). The majority of people with intellectual disabilities were happy to take medication for their pain when given to them by staff, but only 18% stated they would take medicine if they were in pain when alone at home, suggesting that independent use of medication was low.

#### 3.3.2 Use of medication

Along with exploring people with intellectual disabilities' understanding of what medication they take and why, three studies explored understanding about how medication should be used. Davis et al. (2014) presented six comments from clinic doctors regarding inhaler use written during clinic attendance for patients with asthma. All comments indicated that the patients lacked knowledge regarding their asthma prescription medication. One patient stated being “unsure how to use puffer” and another “uses inhaler without spacer; needs constant reminding.” However, only six comments were presented out of the 125 available asthma records (hard copy) for people with asthma and intellectual disabilities so an assumption cannot be made that the lack of knowledge was evident across all patients. Davis et al. (2016) extended these findings by asking people with intellectual disabilities about their use of asthma medication. Although the interviews indicated that some participants had not been shown how to use their prescribed inhaler, others were knowledgeable about using different inhalers in different situations, for example, “this one I have if I'm short of breath,” and appeared to understand the significance of using their inhaled medications.

Flood and Henman (2015) conducted a case study with one man with mild intellectual disabilities and diabetes aged between 30 and 40 years. Although the participant appeared to understand what medication he was on and was able to describe how he administers his insulin, he did not store some of his medication (insulin or glucagon) according to the manufacturers' directions.

#### 3.3.3 Decision making

Only two studies presented information about the decision to take medication. The case study participant in Flood and Henman (2015) stated that he took his tablets because he did not “want to die,” suggesting that he understood the purpose of the medication and could use this to make a decision about whether to take it. Most participants in Walmsley et al. (2016) reported having the decision to use contraception made for them, with the exception of two women who appeared to be making proactive decisions about their contraception.

#### 3.3.4 Factors affecting medication knowledge

One study compared medication knowledge between patients with intellectual disabilities with and without capacity and found that medication knowledge was generally better for those patients with intellectual disabilities who had capacity (Huneke et al., 2012). Capacity was defined as having the capacity to consent for medical treatment and was based on staff report. All participants without capacity demonstrated poor knowledge in all areas (name and purpose of medication, dose schedule, and duration of treatment, advantages and potential disadvantages of treatment, consequences of not taking medication and contraindicated foods and drinks). Participants with capacity demonstrated good knowledge (i.e., defined by the authors as above 50% based on past publications (Arscott, Stenfert Kroese, & Dagnan, 2000; Barat, Andreasen, & Damsgaard, 2001)) of advantages of treatment, dose schedule, purpose of the medication and consequences of not taking the medication. Knowledge of the duration of treatment and the names of medications were rated as poor for those with capacity.

### 3.4 Understanding of prescription medication risks

In comparison with general knowledge of prescription medication, the included articles presented little information regarding participants with intellectual disabilities' understanding of medication risks. Overall, participants were often confused or unaware of adverse effects associated with their prescription medication.

#### 3.4.1 Risks of taking multiple medications

Beacroft and Dodd (2010) found that the majority of participants (70%) said that they would take medication when in pain. However, when the remaining participants were asked why they would not take medication, they expressed confusion and concern about whether the pain medication would react negatively with their regular medication. It is not clear, however, whether the 70%, who said they would take their medication as a result of being in pain, had an understanding of the risks of taking their prescription medication, including potential reactions with other medication.

#### 3.4.2 Adverse effects

Three studies discussed participant's understanding of adverse effects of prescription medication, two of which found largely poor knowledge about disadvantages or adverse effects. All participants in Huneke et al.'s (2012) study demonstrated poor knowledge of the possible disadvantages of their medication. However, those with capacity showed very good knowledge on contraindicated foods and drinks. In Davis et al. (2016), the majority of participants seemed unaware of the adverse effects that could result from their asthma medication, although some participants were able to express some physiological undesirable effects of their inhaler, for example, “I had a bad reaction…my legs were shaking, my arms were shaking.”

The participant in Flood and Henman's (2015) case study appeared to have some knowledge of adverse effects. When discussing his medication with the researcher, he stated he knew that diarrhoea was a side effect of two of his medications. However, potential adverse effects of his other medications were not discussed so it cannot be concluded whether he had knowledge of the adverse effects of all his medication.

#### 3.4.3 Lack of information about risks

Two studies indicated that some people with intellectual disabilities recognized that they did not understand enough about the adverse effects and risks of their prescription medications, and expressed the desire for more information. Only one respondent in Fish et al.'s questionnaire study (2017) stated that the pharmacist mentioned an adverse effect of their medication, and four people (7%) expressed the need for information about adverse effects and risks in order for them to make a decision about medicines. Participants were asked to provide suggestions for improvement on the psychotropic medication education group reported by Sheehan et al. (2017); one participant stated they “wanted to know more about side effects.”

### 3.5 How to improve knowledge of prescription medication in people with intellectual disabilities

Eight out of ten articles in this scoping review reported ways in which knowledge of prescription medication amongst people with intellectual disabilities could be improved. It was commonly suggested, that a wide array of sources of information should be readily available for people with intellectual disabilities including easy‐read leaflets with pictures and/or diagrams and media platforms such as YouTube.

#### 3.5.1 Format of information

Four of the included studies discussed how medication information is presented to people with intellectual disabilities. Davis et al.'s qualitative study (2016) found that the way in which information was conveyed to people with intellectual disabilities using inhaled asthma medications was crucial. Written information was often reported to be a challenge to understand, for example, one participant stated “it would be hard for me to read because I'm almost blind in one eye and I read things back to front.” Some participants reported turning to media platforms, such as YouTube, to find out information regarding their medication (Davis et al., 2016).

Extending this, Fish et al. (2017) found that people would like information presented in ways other than verbally, that is, photos/videos of how to use their medication (*n* = 3), mobile alerts/special alarm as a reminder/timetable (*n* = 4). They also expressed how aids such as hearing loops/braille/sign language and/or interpreters should be utilized when appropriate (*n* = 5).

Easy‐read and pictorial support was specifically mentioned in two of the articles. Walmsley et al. (2016) suggested using illustrated and easy‐read information to help improve practice when explaining contraception to people with intellectual disabilities and in Fish et al. (2017), 66% (*n* = 38) of people with intellectual disabilities wanted an easy‐read leaflet and 17% (*n* = 10) wanted pictures or diagrams to help understand their medicine.

These findings around the use of different formats of information were also echoed by clinicians. When exploring the doctors' comments during clinic attendance, Davis et al. (2014) also found suggestions for the use of non‐verbal information when trying to increase the knowledge of prescription medication in people with intellectual disabilities, for example, “some pictures should be given; copies should be shared between [community organisation] and his parents.”

#### 3.5.2 Educating people with intellectual disabilities

Alongside tailoring medication information to the person with intellectual disabilities' needs, the included studies also emphasized the importance of clinicians checking the person's understanding of what is required and/or what support is available when using the medication (Davis et al., 2016; Fish et al., 2017; Walmsley et al., 2016). Two studies conducted interventions to educate people with intellectual disabilities about their medications. Participants in Ferguson and Murphy's study (2014) received discussion‐based group training sessions tailored to their medications (haloperidol, metformin and sodium valproate). Participants completed questionnaires before the training sessions and two weeks after their final training session to measure the effect of the training and a significant difference between A‐ACQ scores at baseline and post‐intervention was found. Improvements were associated with verbal comprehension, suggesting that those with verbal abilities may benefit more from training. However, not all items on the questionnaire directly asked about medication knowledge, therefore making it difficult to draw specific conclusions about medication knowledge.

Participants in Sheehan et al. (2017) also took part in an intervention aiming to educate people with intellectual disabilities about their medication. Participants completed a psychotropic medication knowledge test before and after the education group. The pre‐ and post‐test scores were similar, and there was no statistically significant difference, suggesting that the intervention was not effective at increasing medication knowledge.

#### 3.5.3 Training health professionals

As well as educating people with intellectual disabilities about their medication, five studies found that it is also important to train and educate health professionals in providing information to people with intellectual disabilities. It was identified that respiratory physicians, asthma educators, GPs and pharmacists are important in training inhaler use in people with intellectual disabilities (Davis et al., 2014). Doctors' notes emphasized the need for staff knowledge and training “please make sure staff are aware of how to use the puffers with spacer,” “buy spacer and teach him” to help people with intellectual disabilities understand how to use their medication.

Fish et al. (2017) explored the level of information regarding medication, provided by both GPs and pharmacists from the perspective of people with intellectual disabilities. Fifty‐five per cent of participants (*n* = 32) commented on receiving helpful information which helped their understanding of medication from their GP such as “showed how to use the medication,” “told everything about my medicine,” “I have a good doctor who will tell me what the medicine is for and will answer any questions I have.” However, 29% of participants (*n* = 17) felt the information from their GP was not helpful due to information not being accessible/understandable (*n* = 7), only being provided with basic information (*n* = 6) or the information only being given to their carer (*n* = 2). In two cases, no information was provided by their GP, for example, “I just get a prescription” and 24% (*n* = 14) of participants reported receiving no information about their medication at all from their pharmacist. This suggests a training need for professionals about how to best provide information to people with intellectual disabilities.

As well as training and educating staff and medical professionals on an individual basis, one study exploring the use of diabetes medication in people with intellectual disabilities suggested the need for groups to work together. Flood and Henman (2015) discussed the need to provide person‐centred accessible information and suggested how pharmacists could, where appropriate, put people with intellectual disabilities in touch with “expert” peers for education and support and to encourage engagement with other healthcare professionals involved in their care.

Walmsley et al. (2016) also suggested involving a third party, that is, carer or family member if the person with intellectual disabilities indicates this might be helpful. However, Davis et al. (2016) and Fish et al. (2017) highlighted that it is important for healthcare professionals to talk directly to the person with intellectual disabilities, when appropriate, rather than talking to the carer or support worker. Two participants in Fish et al. (2017) commented on information being given to their carer by GPs and five stated that their pharmacist speaks to their carer instead of them. Although 10 respondents wanted their support workers/families involved and informed, four participants expressed a preference for the information being explained to them rather than their carer. Davis et al. (2016) revealed the frustration experienced by people with intellectual disabilities when doctors talked to the caregiver rather than directly to them, which affects their understanding of their medication “I always have to ask [name of carer] cause the doctors explain it to her.”

## 4 DISCUSSION

### 4.1 Main findings

The aim of this scoping review was to identify and analyse studies that explored the understanding that people with intellectual disabilities have of their prescribed medication, with a focus on the risks of adverse effects, and how to improve their medication understanding. From the 1,326 articles initially extracted from various databases, 10 studies met the inclusion criteria.

This scoping review brings together findings from studies relevant to current policy and practice regarding medication use in people with intellectual disabilities. Overall, these studies suggest that many people with intellectual disabilities do not fully understand their prescription medication, including a lack of knowledge about medication names, how to take their medication and the associated risks (Beacroft & Dodd, 2010; Davis et al., 2016, 2014; Fish et al., 2017; Huneke et al., 2012; Walmsley et al., 2016). Some studies suggested that people with intellectual disabilities are more likely to understand the purpose of their prescription medication than the potential disadvantages and adverse effects (Davis et al., 2016; Huneke et al., 2012). Some studies showed some people with intellectual disabilities were well informed about their medication (Ferguson & Murphy, 2014; Sheehan et al., 2017; Walmsley et al., 2016), and this could be related to better verbal abilities (Ferguson & Murphy, 2014).

Legally, it is required that all patients with mental capacity, including people with intellectual disabilities, should be effectively informed about the risks of their medicines and should receive information in an accessible format for them to help them make decisions about their treatment (Montgomery v Lanarkshire Health Board (Scotland) 2015; NHS England, 2016). However, this review suggests that people with intellectual disabilities, including those with capacity, often have a lack of understanding about the risks of medication, including confusion about potential reactions with other medications (Beacroft & Dodd, 2010) and lack of knowledge of adverse effects (Davis et al., 2016; Huneke et al., 2012).

### 4.2 Implications

#### 4.2.1 Accessibility and delivery of information

Guidelines for healthcare professionals outline the importance of patient involvement in the decision‐making process and encourage effective communication directly with the patient (NICE, 2012), and the AIS (NHS England, 2016) specifies the responsibility of services to make information understandable and accessible to everyone. Therefore, when involving people with intellectual disabilities in the decision‐making process, it is essential to ensure the information being communicated is understandable and accessible (Adams & Carr, 2017). However, this review suggests that people with intellectual disabilities are not always receiving accessible information about their medication directly from healthcare professionals (Davis et al., 2016; Fish et al., 2017; Flood & Henman, 2015). Although people with intellectual disabilities often want their carers or family members to be informed about their medications and to be present during the consultation, they express frustration when the healthcare professional directs the information solely to the caregiver (Davis et al., 2016; Fish et al., 2017).

General recommendations for effective health promotion includes educating and empowering people with intellectual disabilities (Marks & Heller, 2003). The findings from this scoping review suggest that these recommendations apply to consultations regarding medications and that further training may be needed to support healthcare professionals to directly engage people with intellectual disabilities about their medications and make reasonable adjustments to support them. Promoting awareness of the AIS amongst people with intellectual disabilities and their carers may also help them to let their healthcare professional know when they would prefer different formats of information. Through the Disability Partnership project (Mencap, 2016), accessible resources have been produced for people with intellectual disabilities, carers and pharmacists to support them with medications and help achieve greater empowerment.

Risks associated with non‐prescription medicines and complementary treatments are beyond the scope of this review although consideration should be given to the interactions between non‐prescription medicines and prescription medicines, which can lead to harm and potential relapse. It should be noted that non‐prescription medicines can be bought in retail outlets, petrol stations and over the Internet where there are no pharmacists or doctors on site to provide advice.

#### 4.2.2 Education for people with intellectual disabilities

It is important to consider what can be done to ensure people with intellectual disabilities are supported to access and understand information regarding their medication, and risks of adverse effects. Ferguson and Murphy (2014) found that training sessions to improve medication understanding significantly improved participants' capacity in relation to medication taking. This is promising and illustrates that people with intellectual disabilities can be supported to improve their understanding of their medication and be involved in decisions about their treatment. In contrast, Sheehan et al.'s (2017) education group intervention did not have an effect on medication knowledge. Participants in Sheehan et al.'s study, however, were believed by the authors to overestimate their knowledge and skills at baseline and consequently any improvements were difficult to determine.

In order to promote medication understanding, it is important to take into account the needs of the individual and to provide information in the most accessible way, which may include using pictures/diagrams, easy‐read text, audio, video and braille (Fish et al., 2017; Simpson & Douglas, 2011; Strydom et al., 2001; The Disability Partnership, 2016). This places a particular emphasis on the availability, or creation, of appropriate resources. However, patient information leaflets supplied with dispensed medicines from pharmacies can be difficult to understand (Davis, Wolf, Bass, Middlebrooks, et al., 2006; Davis, Wolf, Bass, Thompson, et al., 2006). Easy‐read medication leaflets are freely available on the Internet, but are of variable quality and often not updated for many years (Adams & Shah, 2016a, 2016b), meaning that healthcare professionals and/or carers may need to adapt more general resources about taking medications to meet the person with intellectual disabilities' needs (Hollins, Carpenter, Bradley, & Egerton, 2017; The Disability Partnership, 2016). Group education aimed at specific conditions may also be appropriate for some people with intellectual disabilities (Ferguson & Murphy, 2014), and in some areas, this could be embedded through workshops run by community learning disability teams.

### 4.3 Strengths and limitations

One of the strengths of this scoping review was the use of the five‐stage framework (Arksey & O'Malley, 2005), which allows for transparency and reproducibility. Another strength is the use of two researchers who independently identified and screened the data and the introduction of a third researcher who was consulted in the case of disagreements. Nine out of the ten studies included within this review collected data from the people with intellectual disabilities themselves. This is important as information from a secondary source such as a carer or support worker may not always accurately reflect the views of people with intellectual disabilities.

Several limitations to this scoping review should also be acknowledged. Firstly, articles were only included if published between 2011 and 2017. This approach was taken to focus on current practice, but it is acknowledged that studies prior to 2011 may also offer additional insights. In the majority of studies, the main aim was not to explore participants with intellectual disabilities' knowledge of medication; therefore, some of the findings reported here are only reflective of a relatively small aspect of the original paper. The majority of the studies were conducted in the UK, and so due to differing policy and practice contexts, these findings may not generalize to other countries. Also, some of the studies had limited sample sizes; for example, Dysch et al. (2012) included four participants and Sheehan et al. (2017) included six participants, and so applying findings to the rest of the population with intellectual disabilities could be problematic. Often the language used in the paper's analyses, for example, “some” of the sample, made it difficult to understand the number of participants whom expressed a particular view.

## 5 CONCLUSIONS

Currently, research regarding the understanding of prescription medications in the intellectual disability population is sparse. This scoping review identified 10 studies addressing this issue. Although levels of knowledge about different aspects of medication use varied, often participants had poor knowledge of their medication, how to use it and what the risks of adverse effects were. The level of knowledge and information provision practices described here is unlikely to meet the guidelines currently in place. This review has indicated recommendations for promoting medication understanding based on empirical research, and future research should evaluate how best to improve medication understanding in people with intellectual disabilities.

## CONFLICT OF INTEREST

The authors report no conflict of interests.

## AUTHOR CONTRIBUTIONS

SM planned and drafted the study, protocol and data extraction chart, and MS, DA and CC contributed to this. MS conducted the search and selection process. MS extracted all data. SM provided advice and guidance on the analysis and interpretation of results. MS produced the first draft of the manuscript. All authors contributed to writing and approved the final draft of the manuscript.
